# How Mighty Are the Mitochondria in Causing Individual Differences in Intelligence?—Some Questions for David Geary

**DOI:** 10.3390/jintelligence8010013

**Published:** 2020-03-17

**Authors:** Robert J. Sternberg

**Affiliations:** Department of Human Development, Cornell University, Ithaca, NY 14853, USA; robert.sternberg@cornell.edu

David Geary (2019) has written a summary of his fascinating *Psychological Review* article on the purported role of the mitochondria in the development of intelligence (Geary 2018). This is extremely interesting work, and also refreshing in setting out in a new direction in a field that has a tendency to keep traveling down the same road, over and over again. I have some questions about the article that I will be eager for David Geary to answer in his general response.

## 1. Correlational Nature of This and Other Biologically-Based Models

First, as I have pointed out before (Sternberg 2020), existing biologically-based models of intelligence tend to be correlational, as does this one as well. I was unable to find in the article—perhaps it exists elsewhere—direct evidence that differences in mitochondrial functioning are actually causal, at a meaningful level, of individual or developmental differences in intelligence. There is good evidence, so far as I can tell, of decreases in mitochondrial functioning causing many of the signs we associate with aging (Sun et al. 2016), but many different biologically-based functions decline with age. Articles then could be (and have been) written about how declines in mitochondrial functioning cause cellular senescence, as well as chronic inflammation and also decline in stem-cell activity. Lots of biological functions show declines that correlate with age. Is there experimental evidence of *causal* effects of differences in mitochondrial functioning, in particular, on individual differences in intelligence in humans or allied species? 

## 2. Individual Versus Age-Related Differences in Intelligence

Second, I was unable to find solid evidence of differences in mitochondrial functioning being causal of individual differences in intelligence for a given chronological age. The causes of individual versus developmental differences need not be the same. For example, the causes of typical declines in fluid intelligence in old age may be different from the causes of decline at, say, age 12 years. The mechanisms of decline probably have not taken effect yet at age 12. What is the empirical evidence that individual differences in mitochondrial functioning are causal of individual differences at any given age level, as opposed to being causal just across age levels? Is there any experimental evidence, if not in humans, then in other animals? 

## 3. The Circular Model

Third, the circular model of effects is interesting but, to me, not altogether convincing. It suggests, I believe incorrectly, a hierarchy rather than a heterarchy of effects. The evidence regarding intelligence, I suggest, is that various kinds of genetic and environmental effects covary and interact with each other. Flynn’s work especially suggests how genes and environments work in tandem to create secular differences in intelligence (Flynn 2012, 2016). Additionally, epigenetic effects have demonstrated convincingly how gene-environment relations are interactive rather than just one-way- from the biological to the environmental (Haier 2019). A strictly hierarchical biological model, of one level being fully contained by another, seems dated. Rather, just as mitochondrial functioning may affect various other things, it seems likely that one’s living environment—including nutrition, environmental pollution, and stress—may affect mitochondrial functioning. The factors are not hierarchical, or nested, but interactive in ways that are difficult to predict.

## 4. The Focus on *g* for Understanding Intelligence

Fourth, I believe that intelligence researchers have focused excessively on *g* in seeking to understand intelligence rather than focusing more broadly on intelligence as adaptation (Sternberg 2019). Geary is himself an evolutionary theorist, so I would hope he would be sensitive to my suggestion that researchers focus more on intelligence in its original definitional sense, as broad adaptation to the environment (Colvin et al. 1921). In terms of evolutionary natural selection, of what good is intelligence if it leads a species to destroy itself through its own innovations? These innovations have caused, among other things, serious global climate change, air pollution, water pollution, depletion of natural resources, and pandemics, leading not only to human deaths but also to the extinction of myriad other species (Fears 2019). Psychometric *g* may underlie diverse human cognitive abilities and correlate with culturally approved measures of “success,” but if *g* leads humans to develop so-called “innovations” that ultimately result in humans’ extinction, were the humans then intelligent? If slow-motion self-destruction is intelligent, what is stupid? And do people, at least some of whom are supposed to be intelligent (at least as judged by their prestigious university degrees) choose leaders who not only accept, but actively encourage and promote such destructive paths? 

Researchers should return to the original definition of intelligence as adaptive and present test problems that require adaptive thinking, such as regarding how individuals and collectivities can address issues such as climate change, pollution, governmental corruption, potential pandemics, and xenophobia. We are pursuing such problems in our current research.
